# Prevalence of *BRCA1* and *BRCA2* mutations in unselected breast cancer patients from medellín, Colombia

**DOI:** 10.1186/1897-4287-12-11

**Published:** 2014-04-17

**Authors:** Julián Esteban Londoño Hernández, Marcia Llacuachaqui, Gonzalo Vásquez Palacio, Juan David Figueroa, Jorge Madrid, Mauricio Lema, Robert Royer, Song Li, Garrett Larson, Jeffrey N Weitzel, Steven A Narod

**Affiliations:** 1 Unidad de Genética Médica, Facultad de Medicina, Universidad de Antioquia, Medellín, Colombia; 2 Women’s College Research Institute, 790 Bay Street, 7th Floor, Toronto, Ontario M5G 1N8, Canada; 3 Hospital Pablo Tobón Uribes, Medellín, Colombia; 4 Clínica Las Américas, Medellín, Colombia; 5 Clínica de Oncología Astorga, Clínica SOM, Medellín, Colombia; 6 City of Hope National Medical Center, Duarte, CA, USA

**Keywords:** Colombia, Breast cancer, Hereditary, *BRCA1*, *BRCA2*

## Abstract

**Background:**

Approximately 5% of all breast cancers can be attributed to a mutation in the *BRCA1* or *BRCA2* gene. The genetic component of breast cancer in Colombia has been, for the most part, studied on cases from the Bogota region. Five different founder mutations were in two studies of breast cancer patients in the Bogota region. It is important that the frequency of mutations be established among unselected cases of breast cancer of other regions of Colombia in order to estimate the genetic burden of this cancer in Colombia and to plan genetic services. The aim of this study was to establish the mutation frequencies of the *BRCA* genes in breast cancer patients unselected for family history or age, from Medellin, Colombia.

**Methods:**

We enrolled 280 unselected women with breast cancer from a large public hospital in Medellin, Colombia. A detailed family history from each patient and a blood sample was obtained and processed for DNA analysis. Mutations in *BRCA1* and *BRCA2* were sought using a combination of techniques including a panel of recurrent Hispanic *BRCA* mutations which consists of fifty *BRCA1* mutations and forty-six *BRCA2* mutations, including the five recurrent Colombian *BRCA* mutations. All mutations were confirmed by direct sequencing.

**Results:**

Genetic testing was successfully completed for 244 of the 280 cases (87%). Among the 244 cases, three deleterious mutations were identified (two in *BRCA1* and one in *BRCA2*) representing 1.2% of the total. The average age of breast cancer in the mutation-positive cases was 34 years. The two *BRCA1* mutations were known founder mutations (3450del4 in exon 11 and A1708E in exon 18). The *BRCA2* mutation was in exon 11 (5844del5) and has not been previously reported in individuals of Colombian descent. Among the three mutation-positive families was a breast cancer family and two families with no history of breast or ovarian cancer.

**Conclusion:**

The frequency of *BRCA* mutations in unselected breast cancer cases from the Medellin region of Colombia is low and is approximately 1.2%.

## Introduction

Mutations in the *BRCA1* and *BRCA2* genes confer high susceptibility to both breast and ovarian cancer. The lifetime risk of breast cancer in women who carry a mutation is estimated to be up to 80%, but the risk may vary according to the specific mutation, by the country of residence, and by the family history [1-5]. In Colombia, breast cancer is the most common cancer and is the second most common cause of death among women [6]. In 2008, approximately 6,655 new cases of breast cancer were diagnosed in Colombia [6]. The age-standardized incidence for breast cancer was estimated to be 31.2 cases of breast cancer per 100,000 per year [6], compared to 83.2 per 100,000 per year in Canada and 127.3 cases per 100,000 per year (whites) in the USA (SEER Registry).

Genetic testing for mutations in *BRCA1* and *BRCA2* has potentially important health implications as physicians could offer risk-reducing options such as prophylactic mastectomy and oophorectomy, tamoxifen, as well as specialized surveillance programs for mutation carriers who have not yet developed cancer, as well as targeted cancer therapies for women with *BRCA*-associated cancer [7-11]. However, genetic testing is commonly available in North America, Europe, Australia and Israel, but is not generally available in South America due to its expense. Genetic testing could be made accessible to women in developing countries if common *BRCA* founder mutations in the two genes can be discovered or if the cost of genetic testing is reduced. Populations with a large proportion of genetically associated cancers attributable to founder mutations include the Ashkenazi Jewish [12,13], Bahamian [14], Polish [15], Mexican American [16-18], and French-Canadians [19]. The presence of founder effects within these ethnic groups has enabled rapid and low cost screening compared to the cost of complete sequencing of both *BRCA* genes [20].

Three previous studies have evaluated the prevalence of *BRCA1* and *BRCA2* mutations in breast cancer cases in the Colombian population. These two studies have described five founder mutations in the Colombian population [21,22]. Another study demonstrated a high frequency of recurrent *BRCA* mutations among ovarian cancer cases in Bogota [23]. Although these studies provide strong evidence for an important role of genetics in the etiology of breast and ovarian cancer in Colombia, they have been limited to cases from the Bogota region. Evaluation of unselected cases from other regions of Colombia may lead to the detection of additional founder mutations and will establish whether low cost panel approaches to implementation of genetic services are feasible in this country. We performed mutation analysis of *BRCA1* and *BRCA2* on unselected patients with breast cancer from the region of Medellin, Colombia.

## Material and methods

### Patient population

We conducted a study on unselected breast cancer patients diagnosed at any age from February 2007 to April 2009, recruited from a single large public hospital in Medellin, Colombia (Hospital Universitario San Vicente de Paúl). In total, 280 patients were approached by the research coordinator to participate in the study during an out-patient visit to the medical oncology clinic, or during a hospital admission. All patients agreed to participate. The research coordinator described the study to the patient and informed her of the implications of genetic testing. After written informed consent the patient was interviewed in person by the research coordinator for her medical and lifestyle history as well her family history of cancer, with specific reference to a history of breast or ovarian cancer. Risk factor questionnaires, family pedigrees, and blood samples were obtained for all 280 participants. Tumor histology, tumor size, lymph node involvement and grade were abstracted from the medical records. Bioethics Committee for Human Research of the University Research Center at Universidad de Antioquia (CBEIH-SIU) in Canada and the Research Ethics Board at the Women’s College Research Institute in Canada approved the protocol.

### Laboratory methods

DNA was extracted from blood samples using Puregene DNA extraction kits at the Unidad de Genética Médica, Universidad de Antioquia in Medellín. An adequate amount of DNA was obtained for 244 of the 280 patients (87%) and a sample of this DNA was sent to the laboratory of Dr. Narod. Mutation analysis took place in the laboratory of Dr. Narod at Women’s College Hospital in Toronto and in the laboratory of Dr. Weitzel at City of Hope National Medical Center in California.

Exon 11 of *BRCA1* and exons 10 and 11 of *BRCA2* were screened by the protein truncation test (PTT). Primer sequences used to amplify overlapping fragments for the PTT were obtained from the Breast Cancer Information Core. PTT was performed using the TNT™ rabbit reticulocyte lysate system (Promega Corporation, Madison WI), involving [^35^S] methionine/cysteine (New England Nuclear, Boston MA) for protein detection. We also tested for the three common mutations, *BRCA1* 185delAG, 5382insC and *BRCA2* 6174delT that are most commonly seen in Ashkenazi Jews and others of eastern European ancestry. These founder mutations were assayed using a rapid multiplex method [24].

DNA samples from study subjects were also tested for a panel of recurrent mutations that is estimated to account for up to 80% of Hispanic *BRCA* mutations [23,25]. The panel consists of fifty *BRCA1* and forty-six *BRCA2* mutations, including the five recurrent Colombian *BRCA* mutations reported in the literature, *BRCA1* (A1708E; 3450delCAAG) and *BRCA2* 3034delACAA; 6076delGTTA; 6503delTT [21,22], distributed across four multiplex PCR reactions and analyzed with Sequenom MassArray technology. *BRCA1* A1708E has also been reported as a recurrent mutation in Spain and among Mexican Americans [16,26]. All deleterious mutations were confirmed by direct DNA sequencing.

## Results

A total of 244 breast cancer patients were tested for *BRCA1* and *BRCA2* mutations using a combination of laboratory techniques. On average, 2.3 years had elapsed between the date of diagnosis and the date of interview. The mean age of the patients at diagnosis was 42 years (range 23–55) and the mean age at interview was 44 years (range 25–60). 35% of the patients were diagnosed before the age of 40 and 98% were diagnosed before the age of 50. Only 66 of the patients (27%) had a first-or second degree relative diagnosed with breast cancer or ovarian cancer at any age.

A mutation was found in 3 of 244 (1.2%) patients; two with a mutation in *BRCA1* (0.8%) and one with a mutation in *BRCA2* (0.4%) (Table 1). All three patients were diagnosed at age 40 or below. In this subgroup, the prevalence of mutations was 3.5%. The average age of diagnosis in the women with a *BRCA* mutation was 34 years, compared to 42 years for the non-carriers.

**Table 1:** ***BRCA1 *** **and *BRCA2 *** **mutations identified in Colombian breast cancer patients**

**Patient**	**Gene**	**Exon**	**Mutation**	**Age of diagnosis**	**Family history in first-second degree relatives**
32562	*BRCA1*	11	3450del4	32	3 Breast Cancers (34, 32, 32)
32531	*BRCA1*	18	A1708E	34	None
32259	*BRCA2*	11	5844del5	37	None

A deleterious mutation was seen in 1 of 29 patients (3.4%) with a history of breast cancer in a first-degree relative and in two of 215 (0.9%) patients with no family history of breast cancer in a first-degree relative.

A history of gastric cancer in a first-or second degree relative was reported for 49 of 244 patients (20.1%). None were identified as a mutation carrier. Only one of the three women with a mutation had a first-degree relative affected with breast cancer. Two of 130 patients (1.5%) without a family history of any cancer were found to carry a mutation. None of the mutation carriers had a family history of cancer other than breast cancer.

## Discussion

The aim of the current study was to estimate the prevalence of *BRCA* mutations in a series of unselected female breast cancer patients from the Medellin region in Colombia. We identified a deleterious mutation in 1.2% (3/244) of the women in the study. Among women diagnosed before age of 40, the mutation prevalence was 3.5%. Genetic testing for *BRCA* mutation is justified in this subgroup of women. Only two (3450del4 and A1708E) of the five reported Colombian founder mutations were identified in this study. Thus, the prevalence rate of recurrent mutations in this study is 0.8% which is lower than the 4.2% rate (32/766) found in the unselected breast cancer cases from Bogota [22] and the 15.6% rate (15/96) found in the unselected ovarian cancer cases from the Bogota region [23].

Given that Colombia is a large, populous country and ethnicity varies from state to state, it is important to investigate the prevalence of mutations in other geographical areas of the country other than Bogota. So far studies have been limited to breast cancer patients and ovarian cancer patients from the Bogota region [21-23]. The prevalence rate of *BRCA1* and *BRCA2* mutations in this study is the lowest reported in South American studies with breast cancer populations unselected for age or family history. The prevalence rates in these South American studies range from 2.3-7.1% in Brazil (2.3%) [27], Colombia (4.2%) [22], Chile (7.1%) [28], Mexico (3.6%) (unpublished) and Venezuela (3.6%) (unpublished). The ages of diagnosis (under the age of 50) of the cases in this study is similar to that reported in these studies. This is not surprising given that in Central and South America the population is young and the majority of women with breast cancer are diagnosed under the age of 50 [29]. All three women with a *BRCA* mutation detected in this study were diagnosed under the age of 40, but none had a substantial family history of the disease.

The *BRCA1* 3450del4 founder mutation accounts for 11.5% of unselected ovarian cancers and represents 73% of *BRCA* mutations [23] and 1.6% of unselected breast cancers in Bogota [22]. The *BRCA1* A1708E founder mutation accounts for 1.3% of unselected breast cancers [22] and 1% of unselected ovarian cancers in Bogota [23]. The latter mutation has been observed frequently in Spain [26] and in other Latin American populations [16]. The difference in mutation prevalence could be due to the fact that the patients from the Bogota and Medellin region might have different ethnicities. The high prevalence of the *BRCA1* 3450del4 founder mutation observed in the ovarian cancer cases suggests that there might be a regional population effect in Bogota and consequently this mutation might not have the same prevalence in other regions of Colombia, though the overall number of mutations detected was too small to draw conclusions.

The *BRCA2* 5844del5 mutation has never been seen before in individuals of Colombian descent, and it has only been seen in seven individuals from African descent in the BIC database. It was included in the HISPANEL because it has been observed in other Latin Americans [16]. The Colombian population is characterized by a high African heterogeneity and possesses an important African, Indigenous, and Spanish influence.

Our study has several limitations. We did not perform full sequencing of *BRCA1* and *BRCA2*, nor did we screen for large rearrangements using multiplex ligation-dependent probe amplification (MLPA) with the exception of the *BRCA1* ex9-12del large rearrangement. This deletion was included in the Hispanic panel employed in this study as this deletion is suggested to be a Mexican founder mutation. Nonetheless, this mutation has not been shown to be present in breast cancer patients from the Bogota region of Colombia [22] and consequently it was not observed in this study. Its absence in both Bogota and Medellin supports the likely geographic origin in Mexico. Even though full sequencing of *BRCA1* and *BRCA2* was not conducted, we employed a series of laboratory techniques that included the HISPANEL panel, which is estimated to account for up to 78% of *BRCA* mutations in Hispanic women [23,25]. This estimate of sensitivity is based on the population in Mexico City; the sensitivity of the HISPANEL panel is currently being assessed in other Hispanic populations. In addition, more than 60% of the coding sequence of *BRCA1* and 50% of the coding sequence of *BRCA2* were screened by the PTT assay. Thus, there might be mutations present in the Colombian population that are not included in these assays.

## Conclusion

In conclusion, we have identified a founder *BRCA* mutation in approximately 1.2% of women with breast cancer in the Medellin region of Colombia. It is our goal to document founder mutations in the various ethnic populations of Central and South America to ultimately create a cost-efficient genetic tool that could identify women at high-risk in these countries for the purpose of cancer control.

## Competing interest

The authors declare that they have no competing interests.

## Authors’ contributions

JELH interviewed the participants, collected information, and helped draft the manuscript. ML^2^ analyzed the research data and wrote the manuscript. GVP participated in the design of the study, coordination, and in the supervision of the accrual of study participants. JDF, JM, and ML^5^ participated in the supervision of the accrual of study participants. RR and SL conducted part of the mutation analysis in Toronto. GL and JNW conducted the mutation analysis using the Hispanic mutation panel in California. SN conceived the study, participated in the study design and coordination and helped draft the manuscript. All authors read and approved the final manuscript. All authors read and approved the final manuscript.

## Authors’ information

Co-first author: Marcia Llacuachaqui

## References

[B1] Antoniou A Pharoah PD Narod S Risch HA Eyfjord JE Hopper JL Loman N Olsson H Johannsson O Borg A Pasini B Radice P Manoukian S Eccles DM Tang N Olah E Anton-Culver H Warner E Lubinski J Gronwald J Gorski B Tulinius H Thorlacius S Eerola H Nevanlinna H Syrjäkoski K Kallioniemi OP Thompson D Evans C Peto J Average risks of breast and ovarian cancer associated with BRCA1 or BRCA2 mutations detected in case series unselected for family history: a combined analysis of 22 studies Am J Hum Genet 2003 72 1117 1130 10.1086/375033 12677558 PMC1180265

[B2] King MC Marks JH Mandell JB New York Breast Cancer Study Group Breast and ovarian cancer risks due to inherited mutations in BRCA1 and BRCA2 Science 2003 302 643 646 10.1126/science.1088759 14576434

[B3] Thompson D Easton D Breast Cancer Linkage Consortium Variation in cancer risks, by mutation position, in BRCA2 mutation carriers Am J Hum Genet 2001 68 410 419 10.1086/318181 11170890 PMC1235274

[B4] Thompson D Easton D Breast Cancer Linkage Consortium Variation in BRCA1 cancer risks by mutation position Cancer Epidemiol Biomarkers Prev 2002 11 329 336 11927492

[B5] Ford D Easton DF Bishop DT Narod SA Goldgar DE Risks of cancer in BRCA1-mutation carriers. Breast cancer linkage consortium Lancet 1994 343 692 695 10.1016/S0140-6736(94)91578-4 7907678

[B6] Globocan 2002 Int Agen Res Cancerhttp://www-dep.iarc.fr/

[B7] Metcalfe KA Snyder C Seidel J Hanna D Lynch HT Narod S The use of preventive measures among healthy women who carry a BRCA1 or BRCA2 mutation Fam Cancer 2005 4 97 103 10.1007/s10689-005-4215-3 15951959

[B8] Narod SA Foulkes WD BRCA1 and BRCA2: 1994 and beyond Nat Rev Cancer 2004 4 665 676 10.1038/nrc1431 15343273

[B9] Warner E Causer PA MRI surveillance for hereditary breast-cancer risk Lancet 2005 365 1747 1749 10.1016/S0140-6736(05)66520-8 15910935

[B10] Weitzel JN Buys SS Sherman WH Daniels AM Ursin G Daniels JR MacDonald DJ Blazer KR Pike MC Spicer DV Reduced mammographic density with use of a gonadotropin-releasing hormone agonist-based chemoprevention regimen in BRCA1 carriers Clin Cancer Res 2007 13 654 658 10.1158/1078-0432.CCR-06-1902 17255289

[B11] Tutt A Robson M Garber JE Domchek SM Audeh MW Weitzel JN Friedlander M Arun B Loman N Schmutzler RK Wardley A Mitchell G Earl H Wickens M Carmichael J Oral poly(ADP-ribose) polymerase inhibitor olaparib in patients with BRCA1 or BRCA2 mutations and advanced breast cancer: a proof-of-concept trial Lancet 2010 376 235 244 10.1016/S0140-6736(10)60892-6 20609467

[B12] Tonin P Weber B Offit K Couch F Rebbeck TR Neuhausen S Godwin AK Daly M Wagner-Costalos J Berman D Grana G Fox E Kane MF Kolodner RD Krainer M Haber DA Struewing JP Warner E Rosen B Lerman C Peshkin B Norton L Serova O Foulkes WD Garber JE Frequency of recurrent BRCA1 and BRCA2 mutations in ashkenazi jewish breast cancer families Nat Med 1996 2 1179 1183 10.1038/nm1196-1179 8898735

[B13] Warner E Foulkes W Goodwin P Meschino W Blondal J Paterson C Ozcelik H Goss P Allingham-Hawkins D Hamel N Di Prospero L Contiga V Serruya C Klein M Moslehi R Honeyford J Liede A Glendon G Brunet JS Narod S Prevalence and penetrance of BRCA1 and BRCA2 gene mutations in unselected ashkenazi jewish women with breast cancer J Natl Cancer Inst 1999 91 1241 1247 10.1093/jnci/91.14.1241 10413426

[B14] Donenberg T Lunn J Curling D Turnquest T Krill-Jackson E Royer R Narod SA Hurley J A high prevalence of BRCA1 mutations among breast cancer patients from the Bahamas Breast Cancer Res Treat 2011 125 591 596 10.1007/s10549-010-1156-9 20838878

[B15] Gorski B Byrski T Huzarski T Jakubowska A Menkiszak J Gronwald J Pluzanska A Bebenek M Fischer-Maliszewska L Grzybowska E Narod SA Lubinski J Founder mutations in the BRCA1 gene in polish families with breast-ovarian cancer Am J Hum Genet 2000 66 1963 1968 10.1086/302922 10788334 PMC1378051

[B16] Weitzel JN Clague J Martir-Negron A Ogaz R Herzog J Ricker C Jungbluth C Cina C Duncan P Unzeitig G Saldivar JS Beattie M Feldman N Sand S Port D Barragan DI John EM Neuhausen SL Larson GP Prevalence and type of BRCA mutations in hispanics undergoing genetic cancer risk assessment in the southwestern united states: a report from the clinical cancer genetics community research network J Clin Oncol 2013 31 210 216 10.1200/JCO.2011.41.0027 23233716 PMC3532393

[B17] Weitzel JN Lagos V Blazer KR Nelson R Ricker C Herzog J McGuire C Neuhausen S Prevalence of BRCA mutations and founder effect in high-risk hispanic families Cancer Epidemiol Biomarkers Prev 2005 14 1666 1671 10.1158/1055-9965.EPI-05-0072 16030099

[B18] Weitzel JN Lagos VI Herzog JS Judkins T Hendrickson B Ho JS Ricker CN Lowstuter KJ Blazer KR Tomlinson G Scholl T Evidence for common ancestral origin of a recurring BRCA1 genomic rearrangement identified in high-risk hispanic families Cancer Epidemiol Biomarkers Prev 2007 16 1615 1620 10.1158/1055-9965.EPI-07-0198 17646271

[B19] Tonin PN Mes-Masson AM Futreal PA Morgan K Mahon M Foulkes WD Cole DE Provencher D Ghadirian P Narod SA Founder BRCA1 and BRCA2 mutations in French Canadian breast and ovarian cancer families Am J Hum Genet 1998 63 1341 1351 10.1086/302099 9792861 PMC1377544

[B20] Narod SA Screening for BRCA1 and BRCA2 mutations in breast cancer patients from Mexico: the public health perspective Salud Publica Mex 2009 51 s191 s196 19967274 10.1590/s0036-36342009000800009

[B21] Torres D Rashid MU Gil F Umana A Ramelli G Robledo JF Tawil M Torregrosa L Briceno I Hamman U High proportion of BRCA1/2 founder mutations in hispanic breast/ovarian cancer families from colombia Breast Cancer Res Treat 2007 103 225 232 10.1007/s10549-006-9370-1 17080309

[B22] Torres D Umaña A Robledo JF Caicedo JJ Quintero E Orozco A Torregrosa L Tawil M Hamman U Briceno I Estudio de factores genéticos para cáncer de mama en Colombia Univ Med Bogotá (Colombia) 2009 50 297 301

[B23] Rodríguez AO Llacuachaqui M Pardo GG Royer R Larson G Weitzel JN Narod SA BRCA1 and BRCA2 mutations among ovarian cancer patients from Colombia Gynecol Oncol 2012 124 236 243 10.1016/j.ygyno.2011.10.027 22044689

[B24] Kuperstein G Jack E Narod SA A fluorescent multiplex-DGGE screening test for mutations in the BRCA1 gene Genet Test 2006 10 1 7 10.1089/gte.2006.10.1 16544996

[B25] Weitzel J Ancestry-informed strategies for genetic cancer risk assessment, screening and prevention: adapting the lessons to underserved latinas 2010 San Antonio, TX: 33rd Annual San Antonio Breast Cancer Symposium

[B26] de la Hoya M Osorio A Godino J Sulleiro S Tosar A Perez-Segura P Fernandez C Rodriguez R Diaz-Rubio E Benitez J Devilee P Caldes T Association between BRCA1 and BRCA2 mutations and cancer phenotype in Spanish breast/ovarian cancer families: implications for genetic testing Int J Cancer 2002 97 466 471 10.1002/ijc.1627 11802208

[B27] Gomes MC Costa MM Borojevic R Monteiro AN Vieira R Koifman S Li S Royer R Zhang S Narod SA Prevalence of BRCA1 and BRCA2 mutations in breast cancer patients from Brazil Breast Cancer Res Treat 2007 103 349 353 10.1007/s10549-006-9378-6 17063270

[B28] Gonzalez-Hormazabal P Gutierrez-Enriquez S Gaete D Reyes JM Peralta O Waugh E Gomez F Margarit S Bravo T Blanco R Diez O Jara L Spectrum of BRCA1/2 point mutations and genomic rearrangements in high-risk breast/ovarian cancer chilean families Breast Cancer Res Treat 2011 126 705 716 10.1007/s10549-010-1170-y 20859677

[B29] Bosetti C Rodriguez T Chatenoud L Bertuccio P Levi F Negri E La Vecchia C Trends in cancer mortality in Mexico, 1981–2007 Eur J Cancer Prev 2011 20 355 363 10.1097/CEJ.0b013e32834653c9 21464718

